# Semantic integration to identify overlapping functional modules in protein interaction networks

**DOI:** 10.1186/1471-2105-8-265

**Published:** 2007-07-24

**Authors:** Young-Rae Cho, Woochang Hwang, Murali Ramanathan, Aidong Zhang

**Affiliations:** 1 Department of Computer Science and Engineering, State University of New York, Buffalo, NY, USA; 2 Department of Pharmaceutical Science, State University of New York, Buffalo, NY, USA

## Abstract

**Background:**

The systematic analysis of protein-protein interactions can enable a better understanding of cellular organization, processes and functions. Functional modules can be identified from the protein interaction networks derived from experimental data sets. However, these analyses are challenging because of the presence of unreliable interactions and the complex connectivity of the network. The integration of protein-protein interactions with the data from other sources can be leveraged for improving the effectiveness of functional module detection algorithms.

**Results:**

We have developed novel metrics, called semantic similarity and semantic interactivity, which use Gene Ontology (GO) annotations to measure the reliability of protein-protein interactions. The protein interaction networks can be converted into a weighted graph representation by assigning the reliability values to each interaction as a weight. We presented a flow-based modularization algorithm to efficiently identify overlapping modules in the weighted interaction networks. The experimental results show that the semantic similarity and semantic interactivity of interacting pairs were positively correlated with functional co-occurrence. The effectiveness of the algorithm for identifying modules was evaluated using functional categories from the MIPS database. We demonstrated that our algorithm had higher accuracy compared to other competing approaches.

**Conclusion:**

The integration of protein interaction networks with GO annotation data and the capability of detecting overlapping modules substantially improve the accuracy of module identification.

## Background

Protein-protein interactions provide useful insights into functional associations between proteins [1]. The current knowledge base of protein-protein interactions has been built from the heterogeneous data sources generated by high-throughput techniques [2-5]. A wide range of graph-theoretic approaches have been employed for detecting functional modules from protein interaction networks. However, they have been limited in accuracy due to the presence of unreliable interactions and the complex connectivity patterns of the networks. The experimental data sets are susceptible to false positives, i.e., some fraction of the putative interactions detected must be considered spurious because they cannot be confirmed to occur in vivo [6]. The complexity of protein interaction networks caused by cross-talk between modules also makes functional module detection challenging.

To resolve the inaccuracy resulting from false connections, other functional knowledge can be integrated into the protein interaction networks. For example, our group [7] and others [8,9] have investigated the integration of gene expression data from microarray experiments to improve functional module identification. However, gene expression data are also susceptible to experimental sources of bias and noise. The correlations of mRNA levels with even cognate protein expression may be modest at best. These factors limit the usefulness of microarray data for assessing the reliability of protein-protein interactions. Gene Ontology (GO) [10] is another useful data source to combine with the protein interaction networks. The GO is currently one of the most comprehensive and well-curated ontology databases in the bioinformatics community. It provides a collection of well-defined biological terms, called GO terms, spanning biological processes, molecular functions and cellular components. The GO has been used to facilitate the analysis of gene expression data [11-13].

In this work, we integrate protein-protein interactions with the information content in the GO annotation database to enhance the modularization of interaction networks. An unweighted protein interaction network can be converted into a weighted graph representation by assigning a weight to each interaction [14]. The weight of each interaction is interpreted as its reliability, i.e., the probability of the interaction being a true positive. We propose two novel metrics to measure the reliability of protein-protein interactions using GO annotation data. Recently, Lubovac *et al*. [15] defined the similarity between two proteins as the average of pairwise term-term similarity values from all GO terms that have the annotation of the proteins. However, this definition underestimates the interaction reliability between two proteins that are annotated on many different GO terms, because the interaction may be arisen from the functionality relevant to the GO terms that are the most similar. Our reliability measurements are unique in that the GO annotations and the interacting patterns of the annotated proteins are both characterized.

A functional module is defined as a maximal set of proteins that are involved in the same biological process or function. Based on the assumption that the members in the same module strongly bind each other, a functional module is described as a sub-network in a protein interaction network. Thus, identifying functional modules can be a graph clustering problem. We present an efficient algorithm to identify functional modules in a protein interaction network. Our algorithm is capable of detecting overlapping modules, whereas most of the previous graph clustering approaches generate disjoint modules with mutually exclusive sets of proteins. Because a molecule generally performs different biological processes or functions in different environments, real functional modules are overlapping. Our modularization algorithm first selects a small number of informative proteins, which work as representatives of modules. Next, it simulates information flow starting from each informative protein through the whole weighted interaction network. The flow then reveals a set of proteins under the influence of the informative protein as a potential functional module. The modules may overlap with each other if two or more informative proteins influence the same proteins. Our experimental results demonstrate that the modules identified by our algorithm are statistically significant in terms of cellular functions.

### Previous graph clustering approaches

Graph clustering approaches can be categorized into three groups based on the underlying methodology: density-based clustering, partition-based clustering and hierarchical clustering. Density-based clustering approaches search for densely connected sub-graphs. A typical example is the maximum clique algorithm [16] for detecting fully connected, complete sub-graphs. To overcome the high stringency imposed by the maximum clique algorithm, relatively dense sub-graphs can be identified rather than complete sub-graphs by either using a density threshold or optimizing an objective density function [16,17]. A variety of algorithms using alternative density functions have been presented [18-21]. Recently, several density-based approaches have attempted to uncover overlapping clusters [22,23]. Density-based clustering methods can detect the groups of proteins densely connected each other in a protein interaction network. However, in a global view, they are not able to partition the whole network, which typically has power-law degree distributions [24], wherein sparsely connected nodes are abundant. Because the sparse connections decrease the density of clusters, the large amounts of sparsely connected nodes are excluded from the clusters generated by density-based methods.

Partition-based clustering approaches explore the partition of a network including all sparsely connected nodes. The Restricted Neighborhood Search Clustering (RNSC) algorithm [25] discovers the best partition using a cost function. It starts with randomly partitioning a network, and iteratively moves the nodes on the border of a cluster to an adjacent cluster to decrease the total cost of clusters. It can finally find the partitions with the lowest cost. A critical drawback of this method is that the knowledge of the exact number of clusters in a network is pre-required.

Hierarchical clustering approaches can be justified for bioinformatics applications because of the hierarchical organization of biological systems [26,27], and do not require prior knowledge of the number of clusters in a network. These methods iteratively merge nodes or recursively divide a graph into two or more sub-graphs. For iteratively merging nodes, the similarity or distance between two nodes or two groups of nodes should be measured [28-30]. The Super Paramagnetic Clustering (SPC) method [16,31] is another example of iterative merging. On the other hand, for recursively dividing a graph, the nodes or edges to be cut should be precisely selected. For example, they can be found using betweenness, the fraction of shortest paths passing through a node or an edge [32,33]. As a disadvantage, the hierarchical clustering approaches are sensitive to noisy data.

Our group recently developed the STM algorithm [34], which differs from the other methods in that a democratic voting algorithm is used to identify cluster representatives. The voting is based on the signal transduction model derived from the Erlang distribution and the network connectivity. However, this approach is not able to unravel the problem of false interactions in protein interaction networks.

### Overlapping sub-network structures

The protein interaction network is represented by an undirected, un-weighted graph *G*(*V*, *E*) with proteins as a set of nodes *V *and interactions as a set of edges *E*. *N*(*v*_*i*_) denotes the neighbors of *v*_*i*_, the set of nodes connected to the node *v*_*i*_. The degree of *v*_*i *_is then equivalent to the number of neighbors of *v*_*i*_, |*N *(*v*_*i*_)|. A functional module in a protein interaction network *G *is described as a sub-network *G' *structured by a set of nodes *V' *where *V' *⊆ *V *and all the edges among the nodes in *V' *. Because a protein can be included in several different protein complexes to perform different functions, functional modules overlap with each other. However, even though the functional modules share the members, they still provide topological significance with dense intra-connections and sparse interconnections among modules in a protein interaction network.

**Figure 1:**
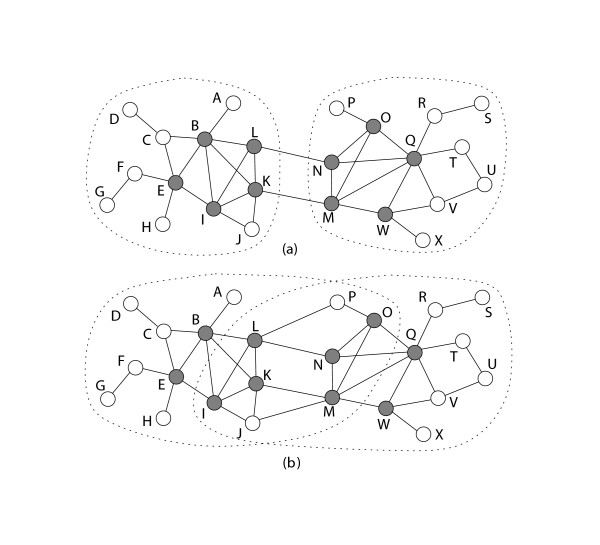
**Examples of disjoint modules and overlapping modules**. The network (a) has two disjoint modules detected by disconnecting two interconnecting edges ⟨*L*, *N*〉 and ⟨*K*, *M*〉. The intra-connection rates of these modules are both 0.89. Each module includes not only core nodes (black color) but also peripheral nodes (white color). The network (b) has two overlapping modules {*A*, *B*, ⋯, *L*, *M*, *N*, *O*, *P*} and {*I*, *J*, *K*, *L*, *M*, ⋯, *W*, *X*}. The intra-connection rates of these modules are both 0.87, whereas those of two disjoint sub-graphs by disconnecting ⟨ *L*, *P*⟩, ⟨*L*, *N*⟩, ⟨*K*, *M*⟩ and ⟨*J*, *M*⟩ are 0.81. The intra-connection rate represents the proportion of the number of connections among the nodes in a module to the number of all connections starting from the nodes.

Figure 1(a) shows an example of disjoint modules in a network. Two disjoint modules, {*A*, *B*, ⋯, *L*} and {*M*, *N*, ⋯, *X*}, are clearly detected by disconnecting two edges ⟨*L*, *N*⟩ and ⟨*K*, *M*⟩. The modules can be characterized by the intra-connection rate, which is the number of connections among the nodes in a module over the number of all connections starting from the nodes. The two modules in Figure 1(a) have high intra-connection rates, which are both 0.89. Each module contains a combination of highly connected nodes, called core nodes, and also sparsely connected nodes, referred to as peripheral nodes. The core nodes, whose degree is greater than 3, are colored with black, whereas peripheral nodes with white. Although the peripheral nodes lower the density of modules, we expect that they have functional correlations with the closely connected core nodes.

The network in Figure 1(b) was structured by creating two more interconnecting edges ⟨*L*, *P*⟩ and ⟨*J*, *M*⟩ from the network in Figure 1(a). The intra-connection rates of two sets {*A*, *B*, ⋯, *L*} and {*M*, *N*, ⋯, *X*} are both 0.81. In this network, each set can grow through new connections to generate the modules with higher intra-connection rates. For example, the set {*A*, *B*, ⋯, *L*} may add the nodes {*M*, *N*, *O*, *P*} to form a module {*A*, *B*, ⋯, *L*, *M*, *N*, *O*, *P*}. The intra-connection rate of the module is then increased to 0.87. The other set {*M*, *N*, ⋯, *X*} can also add the nodes {*I*, *J*, *K*, *L*} for a higher intra-connection rate. The overlap between the two modules thus includes the nodes {*I*, *J*, *K*, *L*, *M*, *N*, *O*, *P*}.

### Data integration

We measure the reliability of protein-protein interactions by two novel definitions that quantify the functional correlation of two proteins using Gene Ontology (GO) annotations. The first metric is *semantic similarity*. Semantic similarity has been used in Information Science to evaluate the similarity between two concepts in a taxonomy [35], and we applied it to protein-protein interactions to estimate the similarity between two proteins. We define an annotation size of a GO term as the number of annotated proteins on the GO term. The semantic similarity between two proteins is then calculated based on the annotation size of the GO term, on which both proteins are annotated. According to the transitivity property of GO annotation, if a protein *x *is annotated on a GO term *g*_*i*_, it is also annotated on the GO terms on the path from *g*_*i *_to the root GO term in the GO structure. Thus, the proportion of the annotation size of a GO term to the total number of annotated proteins can quantify the specificity of the GO term. If two proteins are annotated on a more specific GO term, then they are functionally more similar. See Methods for more details of the semantic similarity.

The other metric is called *semantic interactivity*. The semantic interactivity is derived by combining the GO annotation data with the connectivity in a protein interaction network. Suppose a protein *x *is annotated on a GO term *g*_*i *_and a protein *y *is annotated on a GO term *g*_*j*_. If a large proportion of interacting partners of *x *appears in the annotation of *g*_*j *_and a large proportion of interacting partners of *y *appears in the annotation of *g*_*i*_, then *x *and *y *are likely to interact with each other. If *x *and *y *are annotated on the same GO term *g*_*i*_, then the semantic interactivity increases when more interacting partners of *x *and *y *are included in the annotation of *g*_*i*_. See Methods for more details of the semantic interactivity.

We assign the reliability of each protein-protein interaction, measured by semantic similarity and semantic interactivity, to the corresponding edge as a weight, and build a weighted interaction network integrated with the functional information from the GO database.

### Flow-based modularization algorithm

In our earlier study [7], we proposed the flow-based modularization approach to identify overlapping functional modules in a protein interaction network. The input is a weighted interaction network. The modularization process consists of three phases: informative protein selection, flow simulation to detect preliminary modules and a post-process to merge similar preliminary modules.

#### Informative protein selection

In Phase 1, informative proteins are selected based on the weighted degree *d*_*w*_(*x*) of the proteins, which is defined as the sum of the weights of the edges between the node *x *and its neighbors:

dw(x)=∑y∈N(x)w(x,y),
 MathType@MTEF@5@5@+=feaafiart1ev1aaatCvAUfKttLearuWrP9MDH5MBPbIqV92AaeXatLxBI9gBaebbnrfifHhDYfgasaacH8akY=wiFfYdH8Gipec8Eeeu0xXdbba9frFj0=OqFfea0dXdd9vqai=hGuQ8kuc9pgc9s8qqaq=dirpe0xb9q8qiLsFr0=vr0=vr0dc8meaabaqaciaacaGaaeqabaqabeGadaaakeaacqWGKbazdaWgaaWcbaGaem4DaChabeaakiabcIcaOiabdIha4jabcMcaPiabg2da9maaqafabaGaem4DaCNaeiikaGIaemiEaGNaeiilaWIaemyEaKNaeiykaKcaleaacqWG5bqEcqGHiiIZcqWGobGtcqGGOaakcqWG4baEcqGGPaqkaeqaniabggHiLdGccqGGSaalaaa@4534@

where *w*(*x*, *y*) is the weight of the edge between *x *and *y*. Because the weights are obtained from biological knowledge, the weighted degree of a node includes the factors related to the topological significance in the network and biological importance of the corresponding protein. The number of informative proteins selected is a user-dependent parameter in this algorithm.

#### Flow simulation

Phase 2 simulates the flow starting from each informative protein. The flow simulation is based on the concept that the functional information of a protein *s *flows through every possible path in a weighted network. We can thus quantify how much a protein *s *functionally influences other proteins in a network.

As notations, the flow *f*_*s*_(*x *→ *y*) represents the amount of influence of *s *that travels from *x *to *y*, and *inf*_*s*_(*y*) is the amount of influence of *s *on *y*. The algorithm begins with assigning the weighted degree *d*_*w*_(*s*) to each informative protein *s *as an initial amount of influence *inf*_*s*_(*s*), whereas 0 to all non-informative proteins. For each informative protein *s*, the initial amount of influence of *s*, *inf*_*s*_(*s*), is delivered into each neighbor *y *of *s *as being reduced by the weight of the corresponding edge. Thus, the initial flow *f*_*s *_of the influence of *s *is defined as:

*f*_*s*_(*x *→ *y*) = *w*(*s*, *y*)·*inf*_*s*_(*S*)

for each *y ∈ N*(*s*), where 0 ≤ *w*(*s*, *y*) ≤ 1. The amount of influence of *s *on a protein *y*, *inf*_*s*_(*y*), is updated by adding the sum of all incoming flow *f*_*s *_to *y *from its neighbors.

infs(y)=∑x∈N(y)fs(x→y).
 MathType@MTEF@5@5@+=feaafiart1ev1aaatCvAUfKttLearuWrP9MDH5MBPbIqV92AaeXatLxBI9gBaebbnrfifHhDYfgasaacH8akY=wiFfYdH8Gipec8Eeeu0xXdbba9frFj0=OqFfea0dXdd9vqai=hGuQ8kuc9pgc9s8qqaq=dirpe0xb9q8qiLsFr0=vr0=vr0dc8meaabaqaciaacaGaaeqabaqabeGadaaakeaaieGacqWFPbqAcqWFUbGBcqWFMbGzdaWgaaWcbaGaem4CamhabeaakiabcIcaOiabdMha5jabcMcaPiabg2da9maaqafabaGaemOzay2aaSbaaSqaaiabdohaZbqabaGccqGGOaakcqWG4baEcqGHsgIRcqWG5bqEcqGGPaqkaSqaaiabdIha4jabgIGiolabd6eaojabcIcaOiabdMha5jabcMcaPaqab0GaeyyeIuoakiabc6caUaaa@4A85@

In the case of initial flow, *f*_*s*_(*s *→ *y*) is equivalent to *inf*_*s*_(*y*) because all the nodes except *s *have 0 as an initial amount of influence of *s*. The influence of *s *on *y *then traverses all connected edges in the network by the flow defined as:

fs(y→z)=w(y,z)⋅infs(y)|N(y)|,
 MathType@MTEF@5@5@+=feaafiart1ev1aaatCvAUfKttLearuWrP9MDH5MBPbIqV92AaeXatLxBI9gBaebbnrfifHhDYfgasaacH8akY=wiFfYdH8Gipec8Eeeu0xXdbba9frFj0=OqFfea0dXdd9vqai=hGuQ8kuc9pgc9s8qqaq=dirpe0xb9q8qiLsFr0=vr0=vr0dc8meaabaqaciaacaGaaeqabaqabeGadaaakeaacqWGMbGzdaWgaaWcbaGaem4CamhabeaakiabcIcaOiabdMha5jabgkziUkabdQha6jabcMcaPiabg2da9iabdEha3jabcIcaOiabdMha5jabcYcaSiabdQha6jabcMcaPiabgwSixpaalaaabaGaemyAaKMaemOBa4MaemOzay2aaSbaaSqaaiabdohaZbqabaGccqGGOaakcqWG5bqEcqGGPaqkaeaadaabdaqaaiabd6eaojabcIcaOiabdMha5jabcMcaPaGaay5bSlaawIa7aaaacqGGSaalaaa@51D9@

where the edge ⟨*y*, *z*〉 ∈ *E *and 0 ≤ *w*(*y*, *z*) ≤ 1. The algorithm repeatedly sums up the amount of all incoming influence of *s *on each node using Formula 3 and passes the influence through all connected edges with Formula 4. The influence passing through an edge is reduced according to the weight. If the weight is close to 0, then the influence is quickly reduced. In contrast, if an edge ⟨*x*, *y*⟩ is fully reliable, i.e.,*w*(*x*, *y*) = 1, then *inf*_*s*_(*x*) can be transferred to *y *intact.

The algorithm also accumulates all the previous amounts of influence on each node during the flow simulation. The accumulated amount of influence of *s *on a node *x *is a major factor to determine how likely *s *and *x *are to be included in the same functional module. Since the flow visits all the nodes through every possible path, densely connected nodes close to an informative protein *s *generally have larger amount of influence of *s *than sparsely connected nodes.

The flow in a path stops if it reaches a minimum threshold. The flow simulation starting from an informative protein *s *terminates when there is no more flow in the network. A preliminary module is then created with a set of proteins under the accumulated influence of *s*. Simulating the flow from all informative proteins generates a set of preliminary modules that can potentially overlap.

#### Post-process

Phase 3 is a post-processing step that merges similar preliminary modules to produce final modules. The similar preliminary modules result from the functional closeness of two or more informative proteins because an informative protein works as the representative of a preliminary module in terms of functionality. The similarity *S*(*M*_*s*_, *M*_*t*_) between two modules *M*_*s *_and *M*_*t *_is measured by the weighted interconnectivity defined as:

S(Ms,Mt)=∑x∈Ms,y∈Mtc(x,y)min⁡(|Ms|,|Mt|),
 MathType@MTEF@5@5@+=feaafiart1ev1aaatCvAUfKttLearuWrP9MDH5MBPbIqV92AaeXatLxBI9gBaebbnrfifHhDYfgasaacH8akY=wiFfYdH8Gipec8Eeeu0xXdbba9frFj0=OqFfea0dXdd9vqai=hGuQ8kuc9pgc9s8qqaq=dirpe0xb9q8qiLsFr0=vr0=vr0dc8meaabaqaciaacaGaaeqabaqabeGadaaakeaacqWGtbWucqGGOaakcqWGnbqtdaWgaaWcbaGaem4CamhabeaakiabcYcaSiabd2eannaaBaaaleaacqWG0baDaeqaaOGaeiykaKIaeyypa0ZaaSaaaeaadaaeqaqaaiabdogaJjabcIcaOiabdIha4jabcYcaSiabdMha5jabcMcaPaWcbaGaemiEaGNaeyicI4Saemyta00aaSbaaWqaaiabdohaZbqabaWccqGGSaalcqWG5bqEcqGHiiIZcqWGnbqtdaWgaaadbaGaemiDaqhabeaaaSqab0GaeyyeIuoaaOqaaiGbc2gaTjabcMgaPjabc6gaUnaabmaabaWaaqWaaeaacqWGnbqtdaWgaaWcbaGaem4CamhabeaaaOGaay5bSlaawIa7aiabcYcaSmaaemaabaGaemyta00aaSbaaSqaaiabdsha0bqabaaakiaawEa7caGLiWoaaiaawIcacaGLPaaaaaGaeiilaWcaaa@5F8A@

where

c(x,y)={1if x=yw(x,y)if x≠y and 〈x,y〉∈E0otherwise.
 MathType@MTEF@5@5@+=feaafiart1ev1aaatCvAUfKttLearuWrP9MDH5MBPbIqV92AaeXatLxBI9gBaebbnrfifHhDYfgasaacH8akY=wiFfYdH8Gipec8Eeeu0xXdbba9frFj0=OqFfea0dXdd9vqai=hGuQ8kuc9pgc9s8qqaq=dirpe0xb9q8qiLsFr0=vr0=vr0dc8meaabaqaciaacaGaaeqabaqabeGadaaakeaacqWGJbWycqGGOaakcqWG4baEcqGGSaalcqWG5bqEcqGGPaqkcqGH9aqpdaGabeqaauaabaqadiaaaeaacqaIXaqmaeaacqqGPbqAcqqGMbGzcqqGGaaicqWG4baEcqGH9aqpcqWG5bqEaeaacqWG3bWDcqGGOaakcqWG4baEcqGGSaalcqWG5bqEcqGGPaqkaeaacqqGPbqAcqqGMbGzcqqGGaaicqWG4baEcqGHGjsUcqWG5bqEcqqGGaaicqqGHbqycqqGUbGBcqqGKbazcqqGGaaicqGHPms4cqWG4baEcqGGSaalcqWG5bqEcqGHQms8cqGHiiIZcqWGfbqraeaacqaIWaamaeaacqqGVbWBcqqG0baDcqqGObaAcqqGLbqzcqqGYbGCcqqG3bWDcqqGPbqAcqqGZbWCcqqGLbqzcqGGUaGlaaaacaGL7baaaaa@6AF8@

The modules with the highest similarity in Formula 5 are iteratively merged until the highest similarity is less than a merging threshold.

## Results

### Reliability of protein-protein interactions

**Figure 2:**
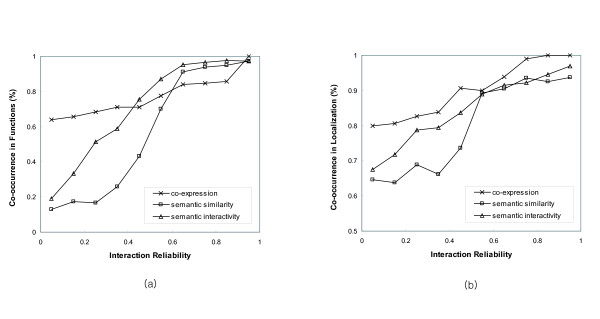
**Functional and localizational co-occurrence of interacting proteins with respect to their reliability**. The proportion of the interacting pairs that co-occur in the same functional and localizational categories, with respect to their reliability measured by semantic similarity, semantic interactivity and co-expression. We used (a) the functional categories on the third level in a hierarchy from MIPS, which includes 170 different functions, and (b) 50 distinct localizational categories from MIPS. The semantic similarity and semantic interactivity provide positive correlations with the co-occurrence in functions and localization.

We extracted the core protein interaction data of Saccharomyces cerevisiae from DIP, the Database of Interacting Proteins [36], and measured their reliability using semantic similarity and semantic interactivity. To validate the reliability for each interaction, we employed the functional and locational categories from the MIPS database [37]. We investigated whether each interacting pair appears in the annotation of the same functional and locational category. Figure 2 illustrates the patterns of the functional and locational co-occurrence of interacting proteins with respect to the reliability measured by semantic similarity, semantic interactivity and co-expression. The co-expression between two genes was computed from the microarray data using the Pearson correlation [7]. See Methods. Based on the reliability for each interacting pair, we divided the interactions into 10 groups. For each group, the proportions of interacting pairs that co-occurred in the same functions and localization were calculated.

For the functional co-occurrence in Figure 2(a), we use the third level categories from the top in a functional hierarchy from MIPS. In general, semantic similarity and semantic interactivity show strong positive correlations with functions. However, the co-expression does not contain sufficient variation of functional co-occurrence, and even interacting pairs with very low co-expression values have relatively high co-occurrence rate (greater than 60%). This result indicates that the functional correlation between two proteins can be better measured by semantic similarity or semantic interactivity than gene co-expression.

The localization is conceptually more general than biological functions. Thus, as shown in Figure 2(b), the co-occurrence of interacting proteins in localizational categories is typically higher than the functional categories, particularly when the values of semantic similarity, semantic interactivity and co-expression are low. Among the three measurements, the semantic interactivity shows the best pattern of positive correlation with localization. Consequently, semantic similarity and semantic interactivity can correctly estimate the reliability of protein-protein interactions.

### Essentiality of informative proteins

As representatives of modules, the informative proteins should be functionally essential. Importantly, Jeong *et al*. [38] have observed that the local connectivity of nodes in a protein interaction network plays a crucial role in cellular functions. The informative proteins were selected based on the weighted degree, which is the metric combined with the local connectivity and the strength of functional relationships.

**Figure 3:**
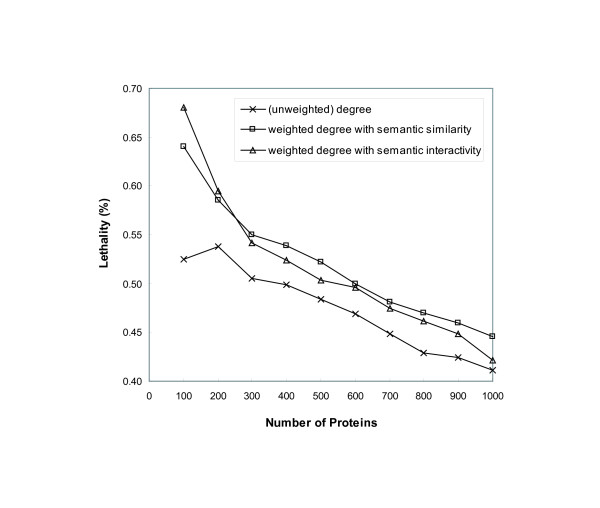
**Lethality of the selected informative proteins**. The proportion of lethal proteins to each set of proteins selected from the highest values of (unweighted) degree and weighted degree using semantic similarity and semantic interactivity. Weighted degree selects more lethal proteins, which are functionally essential in modules, than (unweighted) degree.

To evaluate the functional essentiality of the informative proteins, we employed their lethality information. The lethality is determined from biological experiments in which the protein is knocked out. Protein lethality data were obtained from the MIPS database. We selected the proteins with the highest values of (unweighted) degree and weighted degree using semantic similarity and semantic interactivity, and calculated the proportion of lethal proteins. Figure 3 shows the decreasing pattern of lethality as we have more proteins up to 1000. However, higher proportion of lethal proteins is shown in the sets selected by the weighted degree than the (unweighted) degree. Up to 250 proteins, semantic interactivity selected more lethal proteins than semantic similarity, and fewer lethal proteins when we have more than 250 proteins. This result reflects that the functional essentiality of informative proteins can be determined by both the number of and the strength of functional relationships to other proteins.

### Identification of overlapping modules

We implemented the flow-based modularization algorithm with the core protein interaction data from DIP. As inputs, we used two interaction networks weighted by semantic similarity and semantic interactivity. The algorithm requires two user-dependent parameter values: the number of informative proteins and the minimum amount of flow on a node. The number of modules in an output set depends on the number of selected informative proteins. On the other hand, the minimum amount of flow determines the average size of output modules. By changing the two parameter values, we achieved ten different output sets of modules for each weighted interaction network [see Additional file 1 and 2].

**Figure 4:**
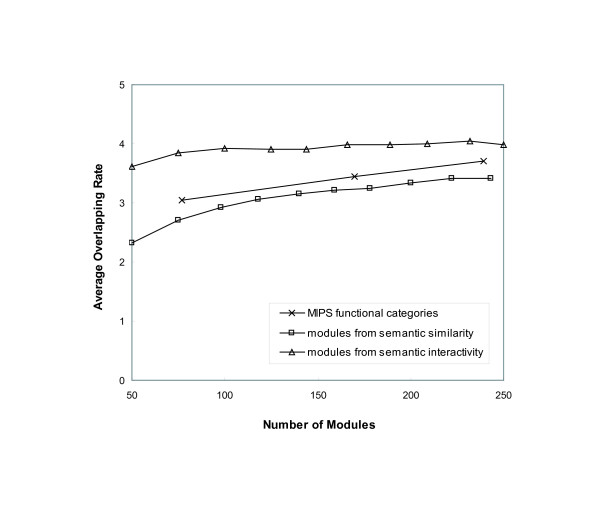
**Protein overlapping patterns of output sets of modules**. The average overlapping rate of proteins with respect to the number of modules in each output set. The flow-based algorithm using semantic similarity and semantic interactivity generated ten different output sets of modules by varying parameter values. Each output set has different number of modules in the range between 50 and 250. The average overlapping rate represents the average number of occurrence of proteins in the modules. The identified modules have a similar overlapping pattern to the MIPS functional categories.

The output modules share a large number of common proteins. To evaluate their overlapping patterns, we counted the number of appearance across different modules for each protein. The average overlapping rates on the sets of identified modules are shown in Figure 4. Each set has the different number of modules in the range between 50 and 250. As expected, the average modules size was greater for the sets with fewer modules. When the protein interaction network was decomposed into more modules, the average overlapping rate was slightly increased. For semantic similarity, the overlapping rate was increased by approximately 10% when the number of generated modules was doubled.

We compared the overlapping rates to those of annotated proteins in functional categories from the MIPS database. The functional categories in MIPS are hierarchically distributed: there are 17 different categories on the top level as the most general functions, and 77, 170 and 239 categories on the second, third and fourth level from the top, respectively. We calculated the average appearance of proteins on the second, third and forth level categories. Figure 4 shows that the average overlapping rate is increased by only 15% despite the three-fold increase in the number of categories between the second level and the fourth. Overall, the modules identified by our algorithm have a similar overlapping pattern when compared to the MIPS functional categories.

### Statistical assessment of the identified modules

To statistically assess the identified modules, we employed the *p*-value from the hypergeometric distribution [17]. We mapped each module to a reference function with the lowest *p*-value, and calculated the negative of *log*(*p*-value). A low *p*-value (equivalently, a high *-log*(*p*-value)) between an identified module and a reference function indicates that the module closely corresponds to the function. The functional categories and their annotations from the MIPS database were used as the reference functions.

We show the performance improvement achieved from semantic integration. The modularization results using semantic similarity and semantic interactivity were compared to the gene co-expression approach we have previously reported [7]. For this comparison, 200 informative proteins were initially selected for each input network, and the minimum flow threshold values were set at 0.4 for semantic similarity, 0.1 for semantic interactivity and 0.03 for genetic co-expression. The average size of output modules was considered to choose the proper value of the minimum flow threshold. Table 1 shows the results of average-*log*(*p*) of the output modules. Overall, weighting with semantic interactivity resulted in the best accuracy of modularization. Besides, both semantic similarity and semantic interactivity outperformed the weighting scheme based on the microarray-derived co-expression.

**Table 1:** Accuracy of output modules by the flow-based method

weighting scheme	modules before post-processing		modules after post-processing	
	
	*-log*(*p*-value)	*f*-measure	*-log*(*p*-value)	*f*-measure
semantic similarity	24.10	0.334	24.42	0.337
semantic interactivity	28.58	0.399	29.05	0.401
genetic co-expression	17.66	0.268	17.42	0.267

The output modules were generated by the flow-based algorithm with 200 informative proteins. The input was the protein interaction networks weighted by three metrics. For each metric, the average values of -*log*(*p*-value) and *f*-measure of the output modules were calculated before and after the post-processing step to merge similar modules.

We also monitored the average -*log*(*p*) of the output modules before and after post-processing. The post-process is the step to merge similar modules after flow simulation. As shown in Table 1, the post-process improved the accuracy of modules generated by the two GO-based weighting methods. The post-process is apparently necessary for the flow-based modularization because two or more informative proteins may represent the same functionality. However, when we use genetic co-expression for weighing, the post-process worsened the accuracy of modules, possibly because modules were merged to generate a less accurate module.

Next, we compared the performance of the flow-based modularization algorithm to three competing state-of-the-art methods: the CFinder algorithm [22] as a density-based method, the betweenness-cut algorithm [32,33] as a hierarchical approach, and the STM algorithm [34]. For each implementation, we selected the parameter values that resulted in the best accuracy. Table 2 shows the parameter values and the results of the output modules. The CFinder algorithm is based on a clique percolation method. Although it is able to find overlapping modules, it detected numerous small-sized modules with a few disproportionally large modules. As a result, the average accuracy of CFinder was lower than the other methods. The betweenness-cut algorithm iteratively disconnects the edges with the highest value of betweenness and recursively proceeds the cutting process in each sub-network. Most of the sparsely connected nodes were included in the output modules. However, because the output modules were disjoint, the betweenness-cut algorithm had a lower accuracy than the flow-based method. The STM algorithm allows the overlap of output modules, but it has much lower rates of overlap than the flow-based method. These results indicate that our flow-based algorithm outperforms other methods in terms of the accuracy of functional module identification.

**Table 2:** Performance comparison of modularization methods

method	number of modules	average size of modules	*-log*(*p*-value)	parameters
flow-based (semantic similarity)	178	46.14	24.42	min flow = 0.4
flow-based (semantic interactivity)	189	40.40	29.05	min flow = 0.1
CFinder	57	17.86	12.32	k = 3
betweenness-cut	57	41.02	17.44	max density = 0.03
STM	60	40.10	13.70	merging thres. = 1.0

The output modules were generated by the flow-based, CFinder, betweenness-cut and STM method. The input was the core protein interaction network from DIP. The performance was evaluated by *p*-value.

**Figure 5:**
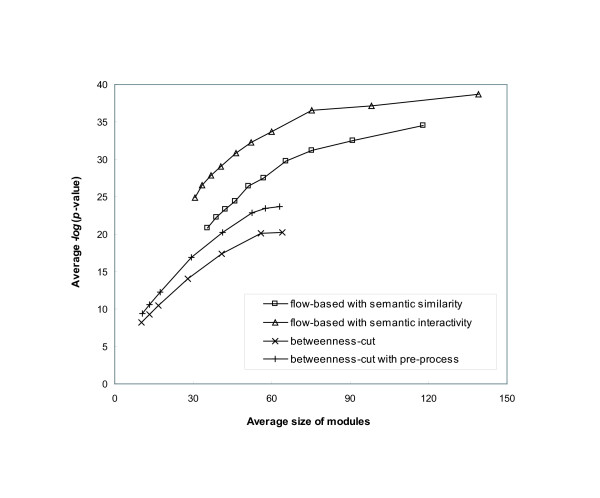
**Statistical significance with respect to the average size of modules**. The average -*log*(*p*-value) of modules with respect to their average size in each output set. Four distinct methods were implemented: the flow-based algorithm using semantic similarity and semantic interactivity, the betweenness-cut algorithm and the betweenness cut algorithm with the pre-processing step to filter out the edges with low semantic similarity. The betweenness-cut algorithm was enhanced by the pre-process, and the flow-based algorithm outperformed the betweenness-cut algorithm.

The *p*-value is highly dependent on the module size. Figure 5 shows the pattern of the average -*log*(*p*) across different sets of output modules produced by varying parameter values for the number of informative proteins and the minimum flow threshold. Although the average value of -*log*(*p*) increased as the average size of modules increased, it converged to approximately 34 and 39 with the semantic similarity and semantic interactivity weighting scheme, respectively. In a similar analysis, we found that the average -*log*(*p*) of the output modules generated by the betweenness-cut algorithm converged to 20 as shown in Figure 5.

False positive interactions in a protein interaction network possibly cause miscalculation of betweenness because the faulty information yields incorrect shortest paths in a network. To resolve this problem, we also implemented the betweenness-cut algorithm with the pre-processing step to filter out potential false positives. We eliminated the edges, whose semantic similarity is less than 0.25, and applied the refined network to the betweenness-cut algorithm. Figure 5 shows the overall accuracy of modules was enhanced by the pre-process. This result implies that the betweenness-cut algorithm is sensitive to false positive interactions. The average *-log*(*p*) converged to approximately 23, which is higher than the result from the betweenness-cut algorithm without pre-processing.

Figure 5 demonstrates that the flow-based modularization algorithm explicitly identified more accurate modules across different output sets than the betweenness-cut algorithm. Using semantic similarity for weighting interactions, we have 70% improvement from the betweenness-cut algorithm and 50% improvement from the betweenness-cut with pre-processing in terms of accuracy when the average size of modules is 60. When larger modules are produced by the flow-based algorithm, the average value of *-log*(*p*) was further increased. These results indicate that when large-sized modules are generated by the flow-based algorithm, the modules are enriched for biological function. Furthermore, overlapping modules obtained by the flow-based algorithm have statistically higher associations with functions than the disjoint modules from partitioning methods.

The subset of the modules identified by our algorithm with high values of -*log*(*p*) are listed with their informative proteins and functions in Table 3. The input network was weighted by semantic interactivity. Some modules have two informative proteins because they were merged during the post-process. It is expected that the informative protein in each module plays a key role in performing the corresponding function.

**Table 3:** Functional modules and informative proteins found in the yeast interaction network

module ID	module size	informative proteins	function	*-log*(*p*-value)
2	81	YLR147c, YGR091w	mRNA processing – splicing	59.88
3	240	YBR160w	mitotic cell cycle	35.37
4	63	YER012w	protein degradation – proteasome	26.48
5	95	YDL140c	mRNA synthesis – general transcription activity	45.23
6	76	YCR093w, YGR134w	mRNA synthesis – transcriptional control	32.23
7	90	YJR022w, YOL149w	mRNA processing – splicing	50.30
13	89	YGR119c	nuclear transport	48.42
18	67	YDR448w	mRNA synthesis – transcriptional control	42.64
19	21	YJR121w	energy generation	28.35
24	50	YGR013w	mRNA processing – splicing	57.60
27	74	YOR181w	actin cytoskeleton	29.85
28	65	YGL172w	RNA transport	44.04
29	30	YLR127c, YDR118w	protein modification – ubiquitination	29.58
39	65	YLR347c	nuclear transport	57.92
47	75	YLR229c	budding and cell polarity	44.52
61	53	YGL092w	structural protein binding	24.01
63	40	YPR181c	vesicular transport – ER to Golgi transport	39.22
65	41	YKL145w	protein modification – proteolytic processing	29.89
71	58	YBL050w	vesicular transport – vesicle fusion	26.75
76	36	YBR088c	DNA repair	23.09
78	48	YLR335w	nuclear transport	49.21
83	46	YJL041w	RNA transport	42.93
89	28	YPR041w	protein synthesis – translation initiation	36.63
95	36	YIL109c	vesicular transport – ER to Golgi transport	41.47
109	52	YER172c	mRNA processing – splicing	53.47
101	24	YGL153w	peroxisome creation	24.57
111	23	YDR244w	peroxisomal transport	26.33
122	62	YHR165c	mRNA processing – splicing	59.90
141	24	YBL023c	DNA synthesis – ori recognition	29.35
151	31	YOR076c	nucleotide metabolism – RNA degradation	31.01
153	39	YDR227w	DNA modification – DNA conformation	24.55
161	28	YLR175w	rRNA processing	21.22
181	33	YOR121w	transmembrane signal transduction	17.46
183	23	YNL102w	DNA synthesis – polymerization	16.01
185	10	YDR016c	cell cycle – chromosomal cycle	14.49

Flow-based modularization algorithm was implemented with 200 informative proteins and 0.1 as a minimum flow threshold. The input network was weighted by semantic interactivity. 35 output modules are listed with their informative proteins, functions and -*log*(*p*-value). Some modules have two informative proteins because they were merged during the post-process.

### Supervised validation of the modules in a hierarchy

To directly compare the identified modules with reference functions, we used a supervised method with recall and precision. Recall measures the tendency of the reference function to match the identified module, whereas precision represents the accuracy of the identified module for matching to the reference function. The *f*-measure is defined as the harmonic mean of recall and precision. The average *f*-measure value of all modules was calculated by mapping each module to the function with the highest *f*-measure value.

The average *f*-measure value of the modules that were generated before and after post-processing are shown in Table 1. Similar to the results from the statistical assessment using *p*-value, the post-process slightly improved the accuracy of modules from our two metrics for interaction reliability measurements, and the semantic interactivity measurement had the best accuracy of modularization.

**Figure 6:**
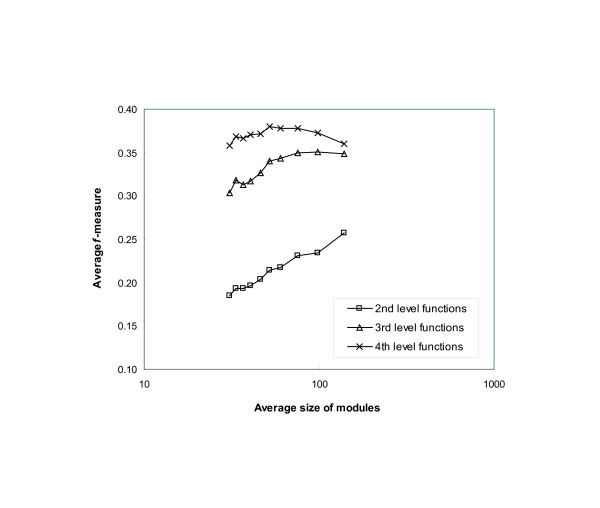
**Average *f*-measure with respect to the average size of modules**. The average *f*-measure value of the modules identified by the flow-based algorithm with respect to their average size in each output set. The output modules were compared to the annotations on the second, third and fourth level categories in the MIPS functional hierarchy. Each plot shows the highest accuracy in a different range of the average size of modules.

We examined whether the flow-based algorithm identifies the various sets of modules on different levels in a functional hierarchy. In the same way to the experiment above, we generated ten different output sets with different parameter values and compared the modules in each set to the annotations on the second, third and fourth level categories in the MIPS functional hierarchy. As shown in Figure 6, when we compared the modules to the functions on the fourth level, which are the most specific functions, we had the highest *f*-measure value. In contrast, we had more mismatches when we compared the modules to large-sized functional categories. In Figure 6, we can also observe that the comparisons to each level of functions provided distinct patterns of accuracy across different output sets. For the second level functions, the modules with the average size of greater than 100 have the highest accuracy. For the third level functions, the modules whose average size is between 70 and 100 have the highest accuracy. Finally, for the fourth level functions, the modules of average size in the range between 40 and 50 have the highest accuracy. Although the results do not strongly support the hierarchical structure, they suggest the possibility of building a hierarchy with the identified modules.

## Conclusion

Experimentally generated protein-protein interaction data includes an enormous amount of false positives. In this paper, we introduced two novel metrics to measure the reliability of interactions. For this measurement, we use the annotations in Gene Ontology (GO), which provides the comprehensive functional information. When we implemented the Betweenness cut algorithm after filtering out the interactions with low semantic similarity values, the overall accuracy of modules is substantially improved. This result strongly appeals the necessity of integrating of functional information for the analysis of protein-protein interaction data.

Most of the previous graph clustering approaches encounter limitations when detecting modules in protein interaction networks. Since they generally generate small-sized dense sub-graphs, they are not adept at identifying hierarchically distributed functional modules. We have developed the flow-based modularization algorithm to identify overlapping modules in a hierarchy. Although the density-based methods, such as CFinder, and the STM algorithm allow overlap among modules, the overlapping rate is very low. However, our algorithm identifies the overlapping modules with high overlapping rate, comparable to the real functional associations of proteins. Since the common proteins in the overlapping modules bridge functional sub-networks, they are topologically and biologically significant.

Another strength of our approach is efficiency. Since we use an index for the adjacency-list representing the input network, the time complexity of the flow simulation is dominated by the index construction, which runs in *O*(*n × m*) where *n *is the number of nodes and *m *is the number of edges. The flow from each informative protein can be simulated in parallel. The total time complexity of the flow-based algorithm depends on the post-process. Suppose we have *k *preliminary modules. We first insert the interconnectivity values for all possible pairs of modules into a heap-based priority queue in *O*(*k*^2 ^log *k*) time. Next, we iteratively merge the most interconnected pair. Each iteration requires *O*(*k *log *k*) time. Hence, the overall time complexity of merging preliminary modules can be solved in *O*(*k*^2 ^log *k*) time where *k *is the number of informative proteins. If we simulate the flow from every node in a network, then the algorithm generates *n *preliminary modules and merges them in *O*(*n*^2 ^log *n*) time where *n *is the number of nodes.

Structuring a modular hierarchy by the flow-based algorithm can be a primary task for deeper analysis of functional relationships among proteins. There are still a large number of functionally uncharacterized proteins in yeast even though it is one of the most well-studied organisms. This study can provide underlying bases for the prediction of functions of the uncharacterized proteins. Moreover, our approach is adaptable to other higher-level organisms because of the efficiency and scalability.

## Methods

### Gene Ontology (GO) structure

The Gene Ontology (GO) database provides GO terms and their relationships. The GO terms are well-defined biological terms, comprising three categories as the most general concepts: biological processes, molecular functions and cellular components. The GO terms are structured by the relationships to each other, such as "is-a" and "part-of". "is-a" and "part-of" represent specific-to-general and part-to-whole relations, respectively. For example, if a GO term *g*_*i *_has a relation "is-a" to *g*_*j*_, then *g*_*i *_is conceptually more specific than *g*_*j*_. A Directed Acyclic Graph (DAG) is then built with the GO terms as a set of nodes and their relationships as a set of directed edges.

### Semantic similarity

Suppose a protein *x *is annotated on *m *different GO terms. *S*_*i*_(*x*) denotes a set of annotated proteins on the GO term *g*_*i*_, whose annotation includes *x*, where 1 ≤ *i *≤ *m*. In the same way, suppose both *x *and *y *are annotated on *n *different GO terms, where *n *≤ *m*. *S*_*j*_(*x*, *y*) denotes a set of annotated proteins on the GO term *g*_*j*_, whose annotation includes *x *and *y*, where 1 ≤ *j *≤ *n*. Then, the minimum size of *S*_*i*_(*x*), min_*i *_|*S*_*i*_(*x*)|, is less than or equal to min_*j *_|*S*_*j*_(*x*, *y*)|.

Suppose the size of annotation represents the number of annotated proteins on a GO term. Using the annotation size of the most specific GO term, on which two proteins *x *and *y *are annotated, we define semantic similarity *S*_*sem*_(*x*, *y*) between *x *and *y *as follows:

Ssem(x,y)=−zlog⁡(min⁡j|Sj(x,y)|/|Sroot|),
 MathType@MTEF@5@5@+=feaafiart1ev1aaatCvAUfKttLearuWrP9MDH5MBPbIqV92AaeXatLxBI9gBaebbnrfifHhDYfgasaacH8akY=wiFfYdH8Gipec8Eeeu0xXdbba9frFj0=OqFfea0dXdd9vqai=hGuQ8kuc9pgc9s8qqaq=dirpe0xb9q8qiLsFr0=vr0=vr0dc8meaabaqaciaacaGaaeqabaqabeGadaaakeaacqWGtbWudaWgaaWcbaGaem4CamNaemyzauMaemyBa0gabeaakiabcIcaOiabdIha4jabcYcaSiabdMha5jabcMcaPiabg2da9iabgkHiTiabdQha6jGbcYgaSjabc+gaVjabcEgaNjabcIcaOmaaxababaGagiyBa0MaeiyAaKMaeiOBa4galeaacqWGQbGAaeqaaOGaeiiFaWNaem4uam1aaSbaaSqaaiabdQgaQbqabaGccqGGOaakcqWG4baEcqGGSaalcqWG5bqEcqGGPaqkcqGG8baFcqGGVaWlcqGG8baFcqWGtbWudaWgaaWcbaGaemOCaiNaem4Ba8Maem4Ba8MaemiDaqhabeaakiabcYha8jabcMcaPiabcYcaSaaa@5DDD@

where

z=1log⁡|Sroot|−log⁡|Smin|.
 MathType@MTEF@5@5@+=feaafiart1ev1aaatCvAUfKttLearuWrP9MDH5MBPbIqV92AaeXatLxBI9gBaebbnrfifHhDYfgasaacH8akY=wiFfYdH8Gipec8Eeeu0xXdbba9frFj0=OqFfea0dXdd9vqai=hGuQ8kuc9pgc9s8qqaq=dirpe0xb9q8qiLsFr0=vr0=vr0dc8meaabaqaciaacaGaaeqabaqabeGadaaakeaacqWG6bGEcqGH9aqpdaWcaaqaaiabigdaXaqaaiGbcYgaSjabc+gaVjabcEgaNnaaemaabaGaem4uam1aaSbaaSqaaiabdkhaYjabd+gaVjabd+gaVjabdsha0bqabaaakiaawEa7caGLiWoacqGHsislcyGGSbaBcqGGVbWBcqGGNbWzdaabdaqaaiabdofatnaaBaaaleaacqWGTbqBcqWGPbqAcqWGUbGBaeqaaaGccaGLhWUaayjcSdaaaiabc6caUaaa@4D19@

*z *is a normalization term using the maximum size of annotation, *S*_*root*_, and the minimum size of annotation, *S*_*min*, _among all GO terms in a DAG structure. If two proteins *x *and *y *are annotated on a more specific GO term than *x *and *z*, then *x *is semantically more similar to *y *than *z*. The semantic similarity *S*_*sem*_(*x*, *y*) can be assigned to the edge between *x *and *y *as a weight.

### Semantic interactivity

Based on the connectivity of a protein *x *in a protein interaction network, we compute the probability *P*(*x*, *y*) that *x *interacts with the annotated proteins on the GO terms, whose annotation includes *y*:

P(x,y)=maxi|Si(y)∩N′(x)||N′(x)|,
 MathType@MTEF@5@5@+=feaafiart1ev1aaatCvAUfKttLearuWrP9MDH5MBPbIqV92AaeXatLxBI9gBaebbnrfifHhDYfgasaacH8akY=wiFfYdH8Gipec8Eeeu0xXdbba9frFj0=OqFfea0dXdd9vqai=hGuQ8kuc9pgc9s8qqaq=dirpe0xb9q8qiLsFr0=vr0=vr0dc8meaabaqaciaacaGaaeqabaqabeGadaaakeaacqWGqbaucqGGOaakcqWG4baEcqGGSaalcqWG5bqEcqGGPaqkcqGH9aqpdaWcaaqaaiabd2gaTjabdggaHjabdIha4naaBaaaleaacqWGPbqAaeqaaOWaaqWaaeaacqWGtbWudaWgaaWcbaGaemyAaKgabeaakiabcIcaOiabdMha5jabcMcaPiabgMIihlqbd6eaozaafaGaeiikaGIaemiEaGNaeiykaKcacaGLhWUaayjcSdaabaWaaqWaaeaacuWGobGtgaqbaiabcIcaOiabdIha4jabcMcaPaGaay5bSlaawIa7aaaacqGGSaalaaa@5190@

where *N'*(*x*) = *N*(*x*) ∪ {*x*}. Since *y *can be annotated on *k *different GO terms, we use the maximum size out of *k *possible sets. If *x *and all of its neighbors are not included in *S*_*i*_(*y*) for any *i*, then *P *(*x*, *y*) is 0. If all of them are included in a set *S*_*i*_(*y*), then *P *(*x*, *y*) is 1. Equation 9 thus satisfies the range of 0 ≤ *P*(*x*, *y*) ≤ 1. We finally measure the semantic interactivity *I*_*sem *_(*x*, *y*) between *x *and *y *using the geometric mean of *P*(*x*, *y*) and *P*(*y*, *x*).

Isem(x,y)=P(x,y)×P(y,x).
 MathType@MTEF@5@5@+=feaafiart1ev1aaatCvAUfKttLearuWrP9MDH5MBPbIqV92AaeXatLxBI9gBaebbnrfifHhDYfgasaacH8akY=wiFfYdH8Gipec8Eeeu0xXdbba9frFj0=OqFfea0dXdd9vqai=hGuQ8kuc9pgc9s8qqaq=dirpe0xb9q8qiLsFr0=vr0=vr0dc8meaabaqaciaacaGaaeqabaqabeGadaaakeaacqWGjbqsdaWgaaWcbaGaem4CamNaemyzauMaemyBa0gabeaakiabcIcaOiabdIha4jabcYcaSiabdMha5jabcMcaPiabg2da9maakaaabaGaemiuaaLaeiikaGIaemiEaGNaeiilaWIaemyEaKNaeiykaKIaey41aqRaemiuaaLaeiikaGIaemyEaKNaeiilaWIaemiEaGNaeiykaKcaleqaaOGaeiOla4caaa@492C@

The semantic interactivity *I*_*sem*_(*x*, *y*) between *x *and *y *can be assigned to the corresponding edge as a weight.

### Expressional correlation

The correlated behaviors in gene expression data, called co-expression, are pertinent to functional associations among molecules. Since the intensities of expressions may include noise due to the microarray experiments, we use the normalized expression data. We then measure the co-expression between two proteins *x *and *y *using the Pearson correlation coefficient *r*:

r=∑i=1n(xi−x¯)(yi−y¯)∑i=1n(xi−x¯)2∑i=1n(yi−y¯)2,
 MathType@MTEF@5@5@+=feaafiart1ev1aaatCvAUfKttLearuWrP9MDH5MBPbIqV92AaeXatLxBI9gBaebbnrfifHhDYfgasaacH8akY=wiFfYdH8Gipec8Eeeu0xXdbba9frFj0=OqFfea0dXdd9vqai=hGuQ8kuc9pgc9s8qqaq=dirpe0xb9q8qiLsFr0=vr0=vr0dc8meaabaqaciaacaGaaeqabaqabeGadaaakeaacqWGYbGCcqGH9aqpdaWcaaqaamaaqadabaWaaeWaaeaacqWG4baEdaWgaaWcbaGaemyAaKgabeaakiabgkHiTiqbdIha4zaaraaacaGLOaGaayzkaaWaaeWaaeaacqWG5bqEdaWgaaWcbaGaemyAaKgabeaakiabgkHiTiqbdMha5zaaraaacaGLOaGaayzkaaaaleaacqWGPbqAcqGH9aqpcqaIXaqmaeaacqWGUbGBa0GaeyyeIuoaaOqaamaakaaabaWaaabmaeaadaqadaqaaiabdIha4naaBaaaleaacqWGPbqAaeqaaOGaeyOeI0IafmiEaGNbaebaaiaawIcacaGLPaaadaahaaWcbeqaaiabikdaYaaaaeaacqWGPbqAcqGH9aqpcqaIXaqmaeaacqWGUbGBa0GaeyyeIuoaaSqabaGcdaGcaaqaamaaqadabaWaaeWaaeaacqWG5bqEdaWgaaWcbaGaemyAaKgabeaakiabgkHiTiqbdMha5zaaraaacaGLOaGaayzkaaWaaWbaaSqabeaacqaIYaGmaaaabaGaemyAaKMaeyypa0JaeGymaedabaGaemOBa4ganiabggHiLdaaleqaaaaakiabcYcaSaaa@62FF@

where *n *is the number of time points for the expressional profiles. The absolute value of *r *can be a weight for the edge between *x *and *y*.

### Hypergeometric *p*-value

As a method for statistical evaluation of modules, we used the *p*-value from the hypergeometric distribution, which is defined as:

P=1−∑i=0k−1(|X|i)(|V|−|X|n−i)(|V|n),
 MathType@MTEF@5@5@+=feaafiart1ev1aaatCvAUfKttLearuWrP9MDH5MBPbIqV92AaeXatLxBI9gBaebbnrfifHhDYfgasaacH8akY=wiFfYdH8Gipec8Eeeu0xXdbba9frFj0=OqFfea0dXdd9vqai=hGuQ8kuc9pgc9s8qqaq=dirpe0xb9q8qiLsFr0=vr0=vr0dc8meaabaqaciaacaGaaeqabaqabeGadaaakeaacqWGqbaucqGH9aqpcqaIXaqmcqGHsisldaaeWbqaamaalaaabaWaaeWaaeaafaqabeGabaaabaWaaqWaaeaacqWGybawaiaawEa7caGLiWoaaeaacqWGPbqAaaaacaGLOaGaayzkaaWaaeWaaeaafaqabeGabaaabaWaaqWaaeaacqWGwbGvaiaawEa7caGLiWoacqGHsisldaabdaqaaiabdIfaybGaay5bSlaawIa7aaqaaiabd6gaUjabgkHiTiabdMgaPbaaaiaawIcacaGLPaaaaeaadaqadaqaauaabeqaceaaaeaadaabdaqaaiabdAfawbGaay5bSlaawIa7aaqaaiabd6gaUbaaaiaawIcacaGLPaaaaaaaleaacqWGPbqAcqGH9aqpcqaIWaamaeaacqWGRbWAcqGHsislcqaIXaqma0GaeyyeIuoakiabcYcaSaaa@57FF@

where |*V*| is the total number of proteins, |*X| *is the number of proteins in a reference function, *n *is the number of proteins in an identified module, and *k *is the number of common proteins between the function and the module. It is understood as the probability that at least *k *proteins in a module of size *n *are included in a reference function of size |*X|*. Low *P *indicates that the module closely corresponds to the function because the network has a lower probability to produce the module by chance.

### Recall, Precision and *f*-measure

Suppose a module *X *is mapped to a functional category *F*_*i*_. Recall, which is also called true positive rate or sensitivity, is the proportion of common proteins between *X *and *F*_*i *_to the size of *F*_*i*_, and precision, which is also called positive predictive value, is the proportion of common proteins between *X *and *F*_*i *_to the size of *X*.

Recall=|X∩Fi||Fi|,
 MathType@MTEF@5@5@+=feaafiart1ev1aaatCvAUfKttLearuWrP9MDH5MBPbIqV92AaeXatLxBI9gBaebbnrfifHhDYfgasaacH8akY=wiFfYdH8Gipec8Eeeu0xXdbba9frFj0=OqFfea0dXdd9vqai=hGuQ8kuc9pgc9s8qqaq=dirpe0xb9q8qiLsFr0=vr0=vr0dc8meaabaqaciaacaGaaeqabaqabeGadaaakeaaieGacqWFsbGucqWFLbqzcqWGJbWycqWGHbqycqWGSbaBcqWGSbaBcqGH9aqpdaWcaaqaamaaemaabaGaemiwaGLaeyykICSaemOray0aaSbaaSqaaiabdMgaPbqabaaakiaawEa7caGLiWoaaeaadaabdaqaaiabdAeagnaaBaaaleaacqWGPbqAaeqaaaGccaGLhWUaayjcSdaaaiabcYcaSaaa@44E8@

and

Precision=|X∩Fi||X|.
 MathType@MTEF@5@5@+=feaafiart1ev1aaatCvAUfKttLearuWrP9MDH5MBPbIqV92AaeXatLxBI9gBaebbnrfifHhDYfgasaacH8akY=wiFfYdH8Gipec8Eeeu0xXdbba9frFj0=OqFfea0dXdd9vqai=hGuQ8kuc9pgc9s8qqaq=dirpe0xb9q8qiLsFr0=vr0=vr0dc8meaabaqaciaacaGaaeqabaqabeGadaaakeaaieGacqWFqbaucqWFYbGCcqWGLbqzcqWGJbWycqWGPbqAcqWGZbWCcqWGPbqAcqWGVbWBcqWGUbGBcqGH9aqpdaWcaaqaamaaemaabaGaemiwaGLaeyykICSaemOray0aaSbaaSqaaiabdMgaPbqabaaakiaawEa7caGLiWoaaeaadaabdaqaaiabdIfaybGaay5bSlaawIa7aaaacqGGUaGlaaa@47CC@

In general, larger size of modules have higher recall because the module *X *is likely to include many members out of *F*_*i*_. As an extreme case, if we generate all the proteins as one module, then we have the maximum value of recall. In contrast, smaller size of modules have higher precision because the members of *X *are likely to be homogeneous in a particular function. As an extreme case, if we generate a single protein as one module, then we have the maximum value of precision. We can thus assess the accuracy of modules with the *f*-measure, which represents the harmonic mean of recall and precision.

### Data sources

The protein-protein interaction data has been accumulated by several high-throughput experiments. We used core interaction data of Saccharomyces cerevisiae from the 2006 version of DIP, the database of interacting proteins [36]. The core interactions have been selected from full data by curative processes based on biological information such as protein sequences and RNA expression profiles [39]. The core data contains 2526 distinct proteins and 5949 interactions.

The gene expression data of Saccharomyces cerevisiae from alpha, CDC15, CDC28, ELU, CLN3 and CLB2 experiments was obtained in SMD [40]. It includes the expression values of 6178 distinct proteins.

The Gene Ontology (GO) Consortium database [10] contains a total of 31890 annotations in the 2006 version. We filtered out excessively specific GO terms. For semantic similarity, we selected the GO terms, which have the annotations of greater than or equal to 10 proteins. For semantic interactivity, we deleted the GO terms with less than 50 annotated proteins. We then chose terminal GO terms, which mean the leaf nodes in the DAG structure of GO. Finally, 129 terminal GO terms with 73.89 of the average size of annotations were extracted.

## Authors' contributions

YRC designed and implemented the data integration and modularization algorithm, analyzed the results and drafted the manuscript. WH partly designed the algorithm and analyzed the results. MR and AZ coordinated the project and revised the final manuscript.

## Supplementary Material

Additional file 1 **Modularization results of the networks weighted by semantic similarity**. Ten different output sets of modules were generated by the flow-based algorithm. The input was the protein interaction network weighted by semantic similarity. To assess the accuracy of modules, the average *f*-measure and the average -*log*(*p*-value) were measured for each output set. Click here for file

Additional file 2 **Modularization results of the networks weighted by semantic interactivity**. Ten different output sets of modules were generated by the flow-based algorithm. The input was the protein interaction network weighted by semantic interactivity. To assess the accuracy of modules, the average *f*-measure and the average -*log*(*p*-value) were measured for each output set. Click here for file
