# Supplementary material for: Semantic integration to identify overlapping functional modules in protein interaction networks
Source: BMC Bioinformatics. 2007 Jul 24;8:265. doi: 10.1186/1471-2105-8-265 (PMC1971074; doi:10.1186/1471-2105-8-265)
Supplement: Additional file 1 — Modularization results of the networks weighted by semantic similarity. Ten different output sets of modules were generated by the flow-based algorithm. The input was the protein interaction network weighted by semantic similarity. To assess the accuracy of modules, the average f-measure and the average -log(p-value) were measured for each output set. [file 1471-2105-8-265-S1.pdf]

Young-Rae Cho, at al.
"Semantic integration to identify overlapping functional modules in protein interaction networks"

Supplementary File 1.

Modularization results of the networks weighted by semantic similarity *before post-processing*

number of informative proteins	min flow threshold	average size of modules	average *f*-measure	average $-log\,(p\text{-}value)$
50	0.180	117.82	0.367	34.51
75	0.220	91.12	0.365	32.55
100	0.260	74.60	0.366	31.32
125	0.300	63.58	0.360	29.71
150	0.333	55.14	0.353	27.47
175	0.367	48.53	0.344	26.05
200	0.400	43.37	0.334	24.10
225	0.425	39.79	0.330	23.16
250	0.450	36.55	0.325	22.06
275	0.480	33.55	0.320	20.72

Modularization results of the networks weighted by semantic similarity *after post-processing*

number of informative proteins	min flow threshold	merging threshold	number of modules	average size of modules	average *f*-measure	average $-log\,(p\text{-}value)$
50	0.180	8.5	50	117.82	0.367	34.51
75	0.220	8.5	75	91.12	0.365	32.55
100	0.260	8.5	98	75.34	0.366	31.22
125	0.300	8.5	118	65.43	0.360	29.77
150	0.333	8.5	140	56.99	0.354	27.55
175	0.367	8.5	159	51.03	0.347	26.37
200	0.400	8.5	178	46.14	0.337	24.42
225	0.425	8.5	200	42.22	0.333	23.37
250	0.450	8.5	222	38.77	0.327	22.27
275	0.480	8.5	243	35.58	0.320	20.82
