# Supplementary material for: Semantic integration to identify overlapping functional modules in protein interaction networks
Source: BMC Bioinformatics. 2007 Jul 24;8:265. doi: 10.1186/1471-2105-8-265 (PMC1971074; doi:10.1186/1471-2105-8-265)
Supplement: Additional file 2 — Modularization results of the networks weighted by semantic interactivity. Ten different output sets of modules were generated by the flow-based algorithm. The input was the protein interaction network weighted by semantic interactivity. To assess the accuracy of modules, the average f-measure and the average -log(p-value) were measured for each output set. [file 1471-2105-8-265-S2.pdf]

Young-Rae Cho, at al.
"Semantic integration to identify overlapping functional modules in protein interaction networks"

Supplementary File 2.

Modularization results of the networks weighted by semantic interactivity *before post-processing*

number of informative proteins	min flow threshold	average size of modules	average *f*-measure	average $-log(p\text{-value})$
50	0.005	139.16	0.373	38.75
75	0.020	98.32	0.381	37.20
100	0.035	75.34	0.388	36.58
125	0.050	60.08	0.400	33.73
150	0.067	50.78	0.403	31.88
175	0.084	44.79	0.397	30.31
200	0.100	39.22	0.399	28.58
225	0.115	35.70	0.391	27.60
250	0.130	32.68	0.393	26.35
275	0.150	29.76	0.378	24.59

Modularization results of the networks weighted by semantic interactivity *after post-processing*

number of informative proteins	min flow threshold	merging threshold	number of modules	average size of modules	average *f*-measure	average $-log(p\text{-value})$
50	0.005	7.0	50	139.16	0.373	38.75
75	0.020	7.0	75	98.32	0.381	37.20
100	0.035	7.0	100	75.34	0.388	36.58
125	0.050	7.0	125	60.08	0.400	33.73
150	0.067	7.0	144	52.08	0.406	32.28
175	0.084	7.0	166	46.22	0.399	30.84
200	0.100	7.0	189	40.40	0.401	29.05
225	0.115	7.0	209	36.76	0.393	27.85
250	0.130	7.0	232	33.53	0.394	26.53
275	0.150	7.0	250	30.66	0.380	24.86
